# Acute Lymphoblastic Leukemia with Infective Endocarditis Presented with Unusual Intracardiac Mass

**DOI:** 10.1155/2017/1528416

**Published:** 2017-05-28

**Authors:** Ali Sadeghpour Tabaei, Leili Koochakzadeh, Mohammadrafie Khorgami, Sepehr Sadeghpour Tabaei

**Affiliations:** ^1^Cardiovascular Department, Rajaie Cardiovascular Medical and Research Center, Iran University of Medical Sciences, Tehran, Iran; ^2^Department of Pediatrics, Children's Medical Center Hospital, Tehran University of Medical Sciences, Tehran, Iran; ^3^Rajaie Cardiovascular Medical and Research Center, Iran University of Medical Sciences, Tehran, Iran; ^4^Shahid Beheshti Medical University, Tehran, Iran

## Abstract

Acute lymphoblastic leukemia (ALL) is a systemic disease that is presented with different symptoms and signs. Cardiac manifestation is rare in ALL, but it is very important and needs appropriate management. It usually presents as leukemic myocardial infiltration and in the presence of cardiac mass comprehensive evaluation for other etiologies is mandatory. We reported on a 6-year-old boy in remission phase of ALL and large cardiac mass in the right atrium with obscure early symptoms and signs, in whom infective endocarditis (IE) was diagnosed and appropriate medical treatment was performed. Because the mass was sustained, surgical resection was considered for the patient.

## 1. Introduction

Acute lymphoblastic leukemia (ALL) results in the malignant proliferation of lymphoid precursor cells of the bone marrow [1, 2] and is the most common cause of pediatric malignancy including 25% of all cancers in children [3]. It has been shown that prognosis would be dependent on the genetic characteristics of ALL, patient's age, the white cell account, clinical status, and the function of major organs. The ALL is a systemic disease that was presented with different symptoms and signs [4]. Although cardiac manifestation is rare in ALL, it is very important and is needed for its appropriate management. The risk of infection in ALL is increased due to several factors including chronic use of indwelling venous catheters, multidrug resistant organisms, malnutrition and failure to thrive, frequent hospitalization, and chemotherapeutic agents [5]. Usually cardiac involvment in ALL manifested as leukemic myocardial infiltration and secondary hemodynamic changes including heart failure were seen due to systemic malignancy effects [6]. In the presence of cardiac mass comprehensive evaluation for other etiologies is mandatory. Intracardiac mass is very uncommon in ALL, but there are many reports of central nervous system (CNS) and testis involvement with lower prevalence in skin, bone, breast, neck, and head [7].

We reported a 6-year-old boy with large cardiac mass in right atrium with obscure early symptoms and signs that infective endocarditis was diagnosed and appropriate treatment was done.

## 2. Case Presentation

A 6-year-old boy admitted in our hospital due to high grade fever, dyspnea, restlessness, and abdominal pain where in outpatient echocardiography the mass in right atrium (RA) was observed and for more evaluation he was referred to our cardiac center. His main disease was acute lymphoblastic leukemia (ALL), diagnosed one year ago after lower limb fracture accident that was refractory to conventional orthopedic therapy. The ALL was in the remission phase with seven courses of previous chemotropic. The history of central venous line insertion was detected. Parents mentioned that their child did not have any other disease in the past. His vital signs include the following: blood pressure, 120/85; pulse rate, 130; respiratory rate, 36; temperature, 38.5. On physical exam patient was pale and undernourished and had shortness of breath, tachypnea, and respiratory distress. On cardiac auscultation the S1 and S2 sounds were loud and grade 4/6 systolic murmur at the left sternal border was heard. The liver was palpated four centimeters below rib. Both knees were swollen with tenderness. There was no significant skin lesion.

### 2.1. Para Clinic

**Figure 1:**
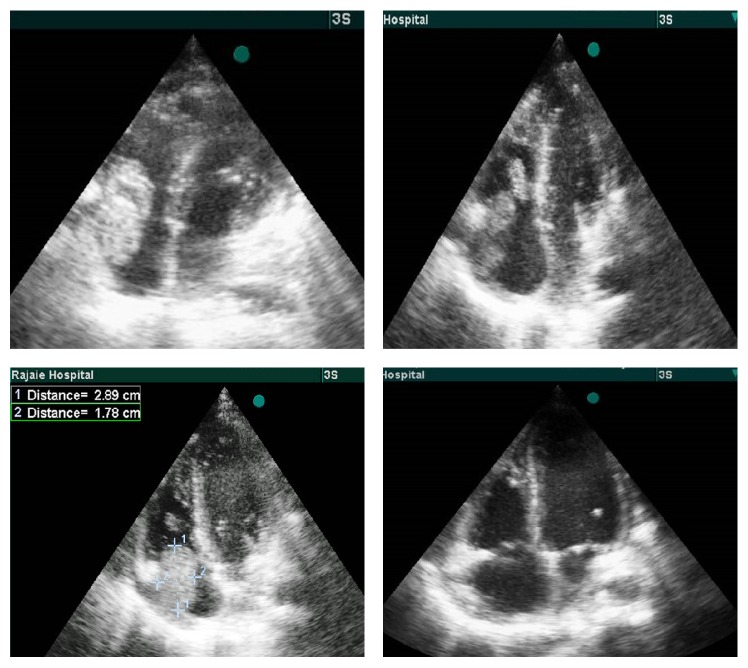
Right atrium vegetation in different view in echocardiography subcostal; four chamber views during systole and diastole. Note mobile position of mass that enters RV during diastole. Last image showed four chamber views after mass resection.

Chest X-ray indicated cardiomegaly and suspicious to pleural effusion in both lungs. Abnormal ECG finding include the following: tall P wave and nonspecific ST-T changes. The laboratory results were the following: Hgb = 8.4 g/dl, WBC = 9300 cells/mm^3^ (Neut: 86.9%, lymph: 5.6%, and mixed: 7.5%), PLT count = 154000, and ESR > 120 mm. During two weeks the WBC increased to 20810 cells/mm^3^, Hgb = 10.8, and PLT count = 155000. Echocardiography finding includes the following: mild right and left ventricle enlargement, mild tricuspid valve regurgitation, mild mitral valve regurgitation, LV ejection fraction = 50%, and RA mass of 2.5 and 3 cm, mobile and attached to tricuspid leaflet in lower border (Figure 1). The CT angiography confirmed the diagnosis of RA mass with sizes 34 and 30 mm; also thromboembolism (PTE) in the right lung and small segment of left lung with bilateral pleural effusion was reported. Perfusion lung scan also indicated PTE in lungs. The blood culture was negative for Bactria and fungi after 48 and 72 hours and one week and all other sampling during hospitalization.

### 2.2. Clinical Course

The diagnosis of infectious endocarditis was considered for child and experimental antimicrobial therapy with vancomycin and meropenem began. Anticoagulant therapy with enoxaparin was started for control of pulmonary thromboemboli (PTE). Rheumatologic consult was done for arthritis and crystalopathy was diagnosed.

**Figure 2:**
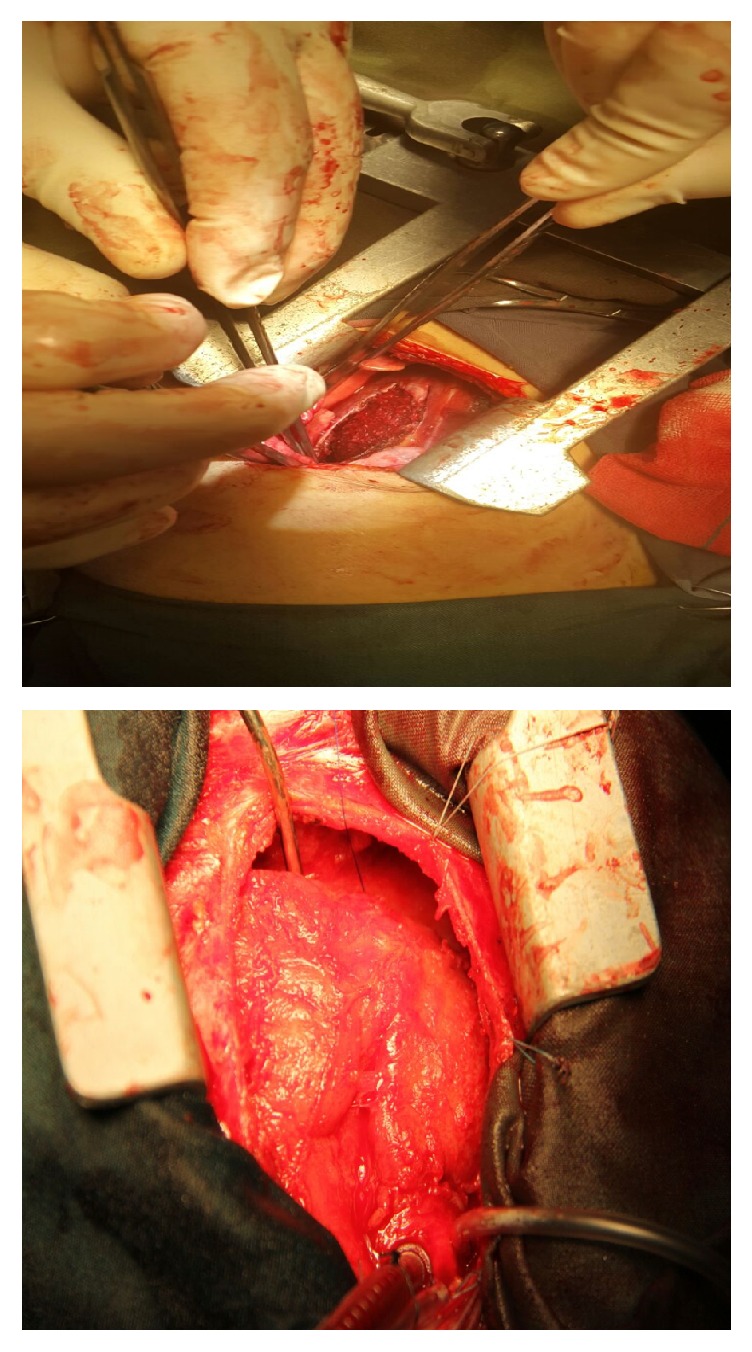
Thickening and inflammation of pericardium.

**Figure 3:**
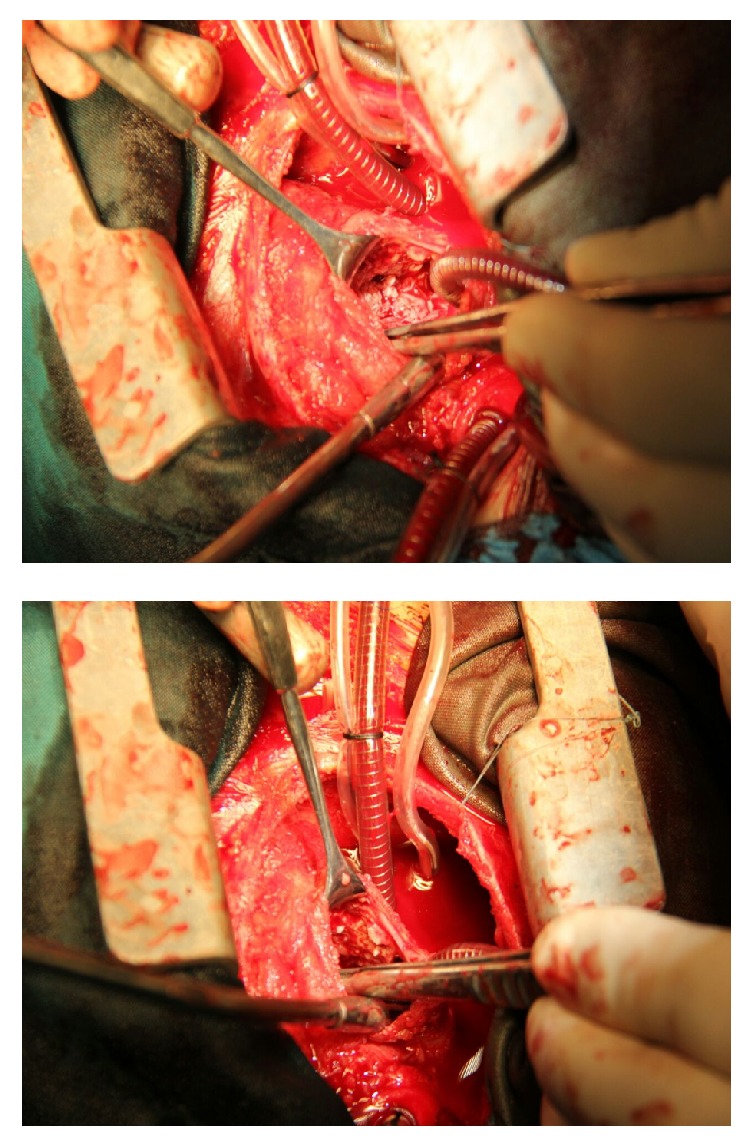
Intracardiac vegetation: one in the tricuspid valve and another in the entrance of SVC to right atrium.

**Figure 4:**
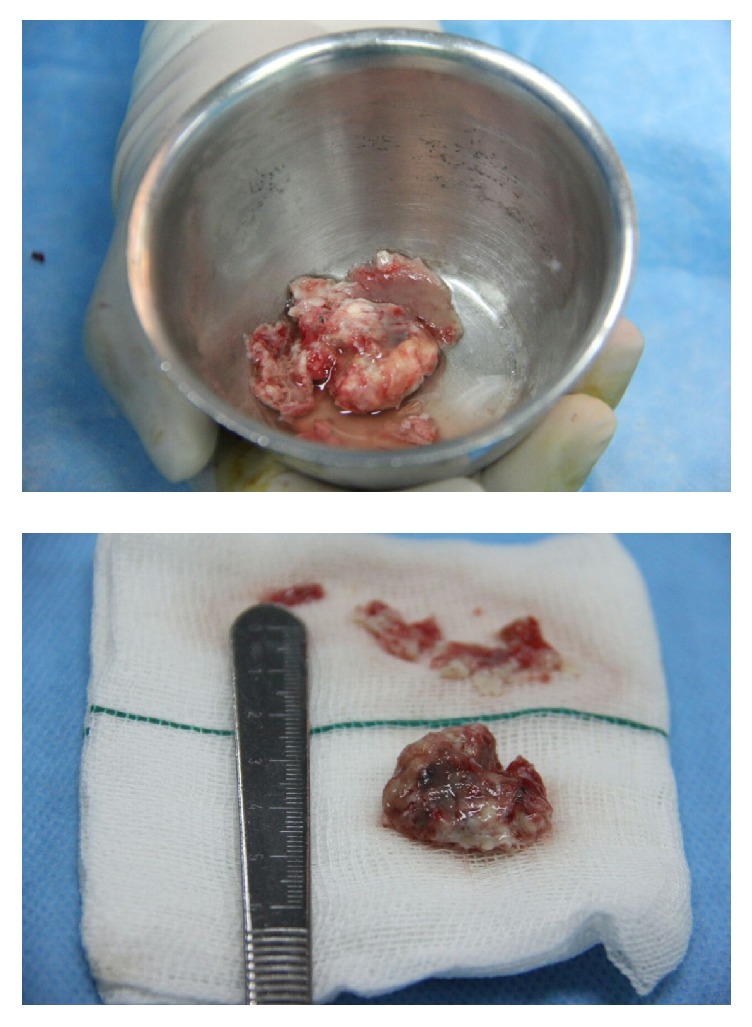
The complete excision of intracardiac mass (vegetation).

Despite antimicrobial therapy the high grade fever continued (>38.5°C) and antifungal treatment with amphotericin added to drug regimen. Serial echocardiography was done but there was no significant decrease in RA mass size. Because response to medical treatment after two weeks was not curative, the surgical mass resection was planned for patient. During the operation after the midsternotomy, the pericardium was thickened and there was adhision to the heart (Figure 2). On cardiopulmonary by-pass and on pump beating heart, after opening RA, there was a lesion in 2 × 2.5 × 3 cm (Figure 2) attached to anterior tricuspid valve (Figure 3). The mass was shaved completely from tricuspid valve and another mass was found near entrance of superior vena cava to right atrium that was resected completely (Figure 4). After surgery pathologic findings showed fibrinoleukocytic exudation with numerous collections resembling gram-positive cocci.

After cardiac operation gradually the general condition improved, fever subsided, and in serial echocardiography there was not any cardiac mass.

## 3. Discussion

Because of nonspecific symptom and sign of infective endocarditis that may overlap with other clinical features in patients with ALL and irreparable burden of late diagnosis and treatment on the final prognosis, exact evaluation for on-time diagnosis of infective endocarditis (IE) is necessary. We encounter RA mass in children with a history of fever, respiratory signs, abdominal pain, and other symptoms and signs of IE during induction chemotherapy. According to Modified Duke Criteria our patients have definitely IE [8]. The pathologic criteria were demonstrated by the presentation of gram-positive cocci and fibrinoleukocytic exudation on histologic examination. Also one major criteria (Oscillating RA mass on tricuspid valve in echocardiography) and three minor criteria include fever with temperature > 38°C, predisposing factor as ALL and major arterial emboli confirmed the clinical criteria.

Although* Viridans* group* streptococci* and staphylococci are common causes of IE in all ages but in immunocompromised patients, gram-negative Bactria is the main substrate for IE [9], although other rare organisms and fungi should be considered [10–14].

In our patient all blood culture that was taken according IE sampling protocol was negative. These include specific culture for fungi and anaerobic microorganisms. Two reasons were explained in patients with IE that blood culture was negative: chronic antibiotic therapy and fastidious organism that in our case seems to be due to recent antibiotic therapy [15]. Kenio et al. reported a case of 15-year-old girl with Down syndrome and ALL; in this patient, source of IE was in the atrium and ventricle and IE was methicillin-sensitive* Staphylococcus aureus* [16].

Unlike fungal endocarditis, gram-positive cocci endocarditis in ALL was reported rarely specially when represented with isolated endocardial RA mass. When the patient does not respond to appropriate medical treatment especially when cardiac vegetation is large, surgical intervention for definite cure is necessary. With regard to the seven courses of chemotherapy in patient and history of central venous catheter (CVC) insertion it seems that indwelling catheter is the source of this event.

In the study of Son et al. in patient with ALL, the IE pathogen was* S. aureus* but without CVC insertion [17]. ALL rarely involves the cardiac chamber; as a result any intracardiac mass in patient with ALL should be considered infective endocarditis.

Schett et al. [10] reported a 60-year-old woman with endocarditis caused by* Aspergillus terreus*, aortic embolization, and splenic infarction. Despite chemotherapy, antibiotic treatment, and cardiac surgery for excision of cardiac vegetation she died because of multiple aortic septic emboli and multiple organ failure.

In conclusion, we recommend performing an exact evaluation for diagnosis of cardiac vegetation in all children with ALL that presented with unexplained fever and other nonspecific signs suspicious of infectious endocarditis, and if the appropriate medical treatment did not elicit response, surgical intervention should be planned as soon as possible. Rational use of antimicrobial agents, prevention of long term inserted indwelling catheter, and continuous monitoring could help to decrease incidence of infectious endocarditis.
